# Data on the impact of subclinical hypothyroidism on clinical outcomes following percutaneous coronary intervention

**DOI:** 10.1016/j.dib.2017.11.080

**Published:** 2017-12-06

**Authors:** Yonggu Lee, Young-Hyo Lim, Jeong-Hun Shin, Jinkyu Park, Jinho Shin

**Affiliations:** a Department of Cardiology, Hanyang University Guri Hospital, Guri City, Kyunggi-do, South Korea; b Division of Cardiology, Department of Internal Medicine, College of Medicine, Hanyang University, Seoul, South Korea

## Abstract

This article contains the data showing the different influence of subclinical hypothyroidism (SCH) on the risk of cardiovascular events after percutaneous coronary intervention (PCI) in various subgroups regarding myocardial infarction, previous PCI, the stent generation, total stent length, the extent of coronary artery disease, diabetes mellitus, obesity, a lipid reduction level and a C-reactive protein level. This article also contains the data showing the association between SCH and the risk of receiving repeat PCI for in-stent restenosis or *de novo* coronary stenosis. The data are supplemental to our original research article titled “Impact of Subclinical Hypothyroidism on Clinical Outcomes Following Percutaneous Coronary Intervention” (Lee et al., 2017) [1].

**Specifications Table**

**Table:** 

Subject area	*Medicine*
More specific subject area	*Cardiology*
Type of data	*Figures and legends*
How data was acquired	*A prospective clinical cohort for patients undergoing PCI*
Data format	*Analyzed (multiple Cox regression analysis and Kaplan-Meier survival analysis results)*
Experimental factors	*The influence of subclinical hypothyroidism on clinical outcome after PCI*
Experimental features	*A prospective cohort study*
Data source location	*Seoul, Republic of Korea*
Data accessibility	*The data are accessible within the article*

**Value of the data**

• Data presented here provide information about the different influence of SCH on clinical outcomes in various subgroups of patients undergoing PCI.
• Data also present whether the influence of SCH on repeat PCI may be different between in-stent restenosis lesion and *de novo* stenotic lesion of coronary arteries.
• These data for the subgroup analysis and the different risk of repeat PCI between in-stent restenosis and *de novo* stenosis may provide a mechanistic insight into the negative influence of SCH on the clinical outcomes following PCI.

## 1 Data

**Fig. 1:**
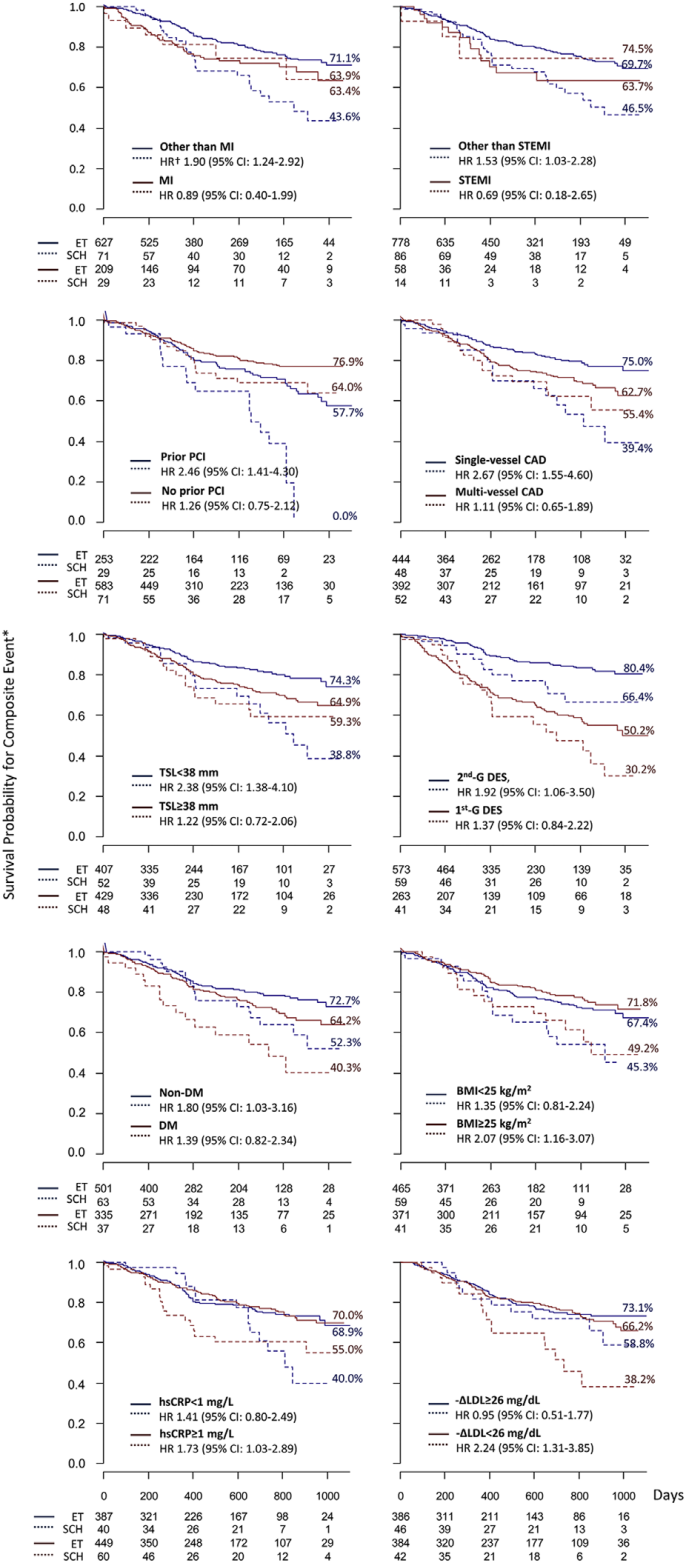
The influence of SCH on the composite event occurrence following PCI in various subgroups. The continuous lines represent the survival estimates in the ET group and the broken lines represent those in the SCH group. The numbers with percentages indicate Kaplan-Meier survival rates at 1000 days of observation. SCH was significantly associated with the composite event in patients with a diagnosis other than MI/STEMI, prior PCI, single-vessel CAD, TSL <38 mm, 2nd-G DES implantation, diabetes mellitus, BMI ≥25 kg/m^2^, hsCRP ≥1.0 mg/l, and -ΔLDL <26 mg/dl. *The composite event is a combination of cardiac death, non-fatal MI and repeat revascularization. †Adjusted for relevant covariates. HR, Hazard ratio; CI, Confidence interval; SCH, subclinical hypothyroidism; ET, euthyroidism; MI, myocardial infarction; STEMI, ST-segment elevation MI; PCI, percutaneous coronary intervention; TSL, total stent length; 2nd-G, Second generation; 1st-G, First generation; DES, Drug-eluting stent; CAD, coronary artery disease; DM, diabetes mellitus; BMI, body mass index; -ΔLDL, low density lipoprotein cholesterol reduction; hsCRP, highly sensitive C-reactive protein.

**Fig. 2:**
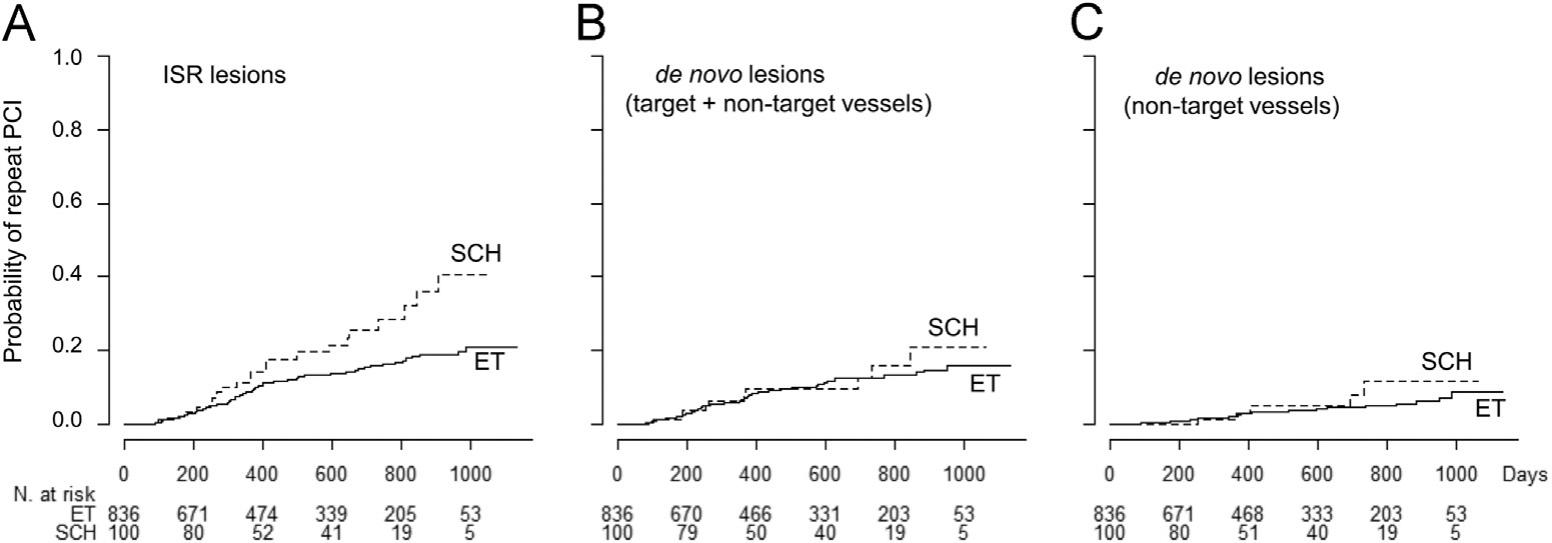
Probabilities of repeat PCIs for ISR lesions (A), *de novo* lesions (B) and non-target vessel *de novo* lesions (C). Repeat PCIs for ISR lesions (A) were more frequently performed in the SCH group than in the ET group, whereas the frequencies of repeat PCIs for *de novo* lesions overall (B) and in non-target vessels (C) were not different between the two groups. PCI, percutaneous coronary intervention; ISR, in-stent restenosis; SCH, subclinical hypothyroidism; ET, euthyroidism.

The data presented include the influence of subclinical hypothyroidism (SCH) on the composite event consisting of cardiac death, non-fatal myocardial infarction (MI) and repeat revascularization in various subgroups (Fig. 1) and the probabilities of repeat percutaneous coronary interventions (PCIs) for in-stent restenosis, overall *de novo* stenotic lesions and *de novo* stenotic lesions in non-target coronary arteries in patients with SCH and those with euthyroidism (ET) (Fig. 2). SCH was associated with the risk of the composite event in patients with a diagnosis other than MI/ST-segment elevation MI (STEMI), prior PCI, single-vessel coronary artery diseases (CAD), total stent length (TSL) <38 mm, second-generation drug-eluting stent (DES) implantation, diabetes mellitus, body mass index (BMI) ≥25 kg/m^2^, highly sensitive C-reactive protein (hsCRP) ≥1.0 mg/l and low-density lipoprotein (LDL) reduction <26 mg/dl (Fig. 1). The risk of repeat PCI was higher in patients with SCH than in patients with ET for in-stent restenosis but not for *de novo* stenosis overall and in non-target coronary arteries (Fig. 2).

## 2 Experimental design, materials and methods

### 2.1 Study design, data collections and definitions

A prospective PCI registry has been operated in the Department of Cardiology, Hanyang University Hospital, Seoul, Republic of Korea since 2009 [2]. Patients enrolled in the registry who underwent PCI with simple balloon angioplasty or stent implantation between March 2010 and December 2013 were consecutively enrolled in this study. Thyroid-stimulating hormone (TSH) levels were measured before the index PCI in all patients. LDL cholesterol levels were measured at the time of index PCI and 3–12 months after PCI. Subclinical hypothyroidism was defined with a serum TSH level ≥4.5 mIU/l and a normal free T4 level (0.7–1.8 mIU/l).

Standardized definitions of clinical events that are frequently used in cardiovascular trials were used in this study, as described by Hicks et al. [3]. Cardiac death included death resulting from acute MI, heart failure or cardiogenic shock. MI was defined as a rise in cardiac troponin I levels to a level at least three times the upper limit of normal, with at least one clinical evidence consistent with myocardial ischemia, including symptoms characteristic of angina, ST-segment changes on electrocardiography and wall motion abnormalities on echocardiography. Repeat revascularization was defined as the performance of balloon angioplasty or stent implantation in the target vessel during the observation period. Repeat revascularization was performed when a lesion with a luminal narrowing ≥70% or functional ischemia was present in the target vessel territory on any diagnostic test. The first clinical event that occurred after the index PCI was used as the patient's clinical event. A composite event was defined as a combination of cardiac death, non-fatal MI and repeat revascularization. Repeat PCI was defined as a PCI that was performed for any causes after index PCI. In-stent restenosis was defined as a re-narrowing >50% at a previously stented site and at the adjacent vascular segments 5 mm proximal and distal to the site.

### 2.2 Statistical analyses

Statistical analyses for this article were performed using statistical software R-3.4.0 and its dedicated packages, rms, KMsurv, survival and descr and are described in detail in the main article [1]. Kaplan-Mayer survival analysis was used to compare event-free survival rates between the ET group and the SCH group in various subsets. To evaluate the associations between SCH and adverse clinical outcomes, multiple Cox-regression analyses were performed with age, sex, current smoking status, diagnosis of STEMI, BMI, lipid profiles, diabetes mellitus, hypertension, hemoglobin levels, log-transformed BNP, renal impairment, left ventricular ejection fraction, TSL, multi-vessel CAD, left main coronary artery intervention, prior PCI and stent generation serving as covariates.

Kaplan-Meier survival curve analyses were performed to compare the risk of repeat PCI for in-stent restenosis, overall *de novo* stenotic lesions and *de novo* stenotic lesion in non-target coronary arteries between the SCH group and the ET group.
